# Frequency specific activity in subthalamic nucleus correlates with hand bradykinesia in Parkinson's disease

**DOI:** 10.1016/j.expneurol.2012.11.011

**Published:** 2013-02

**Authors:** Huiling Tan, Alek Pogosyan, Anam Anzak, Thomas Foltynie, Patricia Limousin, Ludvic Zrinzo, Keyoumars Ashkan, Marko Bogdanovic, Alexander L. Green, Tipu Aziz, Peter Brown

**Affiliations:** a Functional Neurosurgery—Experimental Neurology Group, Nuffield Department of Clinical Neurosciences, John Radcliffe Hospital, University of Oxford, UK; b Sobell Department of Motor Neuroscience and Movement Disorders, UCL Institute of Neurology, UK; c Department of Neurosurgery, Kings College Hospital, Kings College London, UK

**Keywords:** LFP, local field potential, UPDRS, Unified Parkinson's Disease Rating Scale, Basal ganglia, Subthalamic nucleus, Local field potentials, Force decrement, Force release

## Abstract

Local field potential recordings made from the basal ganglia of patients undergoing deep brain stimulation have suggested that frequency specific activity is involved in determining the rate of force development and the peak force at the outset of a movement. However, the extent to which the basal ganglia might be involved in motor performance later on in a sustained contraction is less clear. We therefore recorded from the subthalamic nucleus region (STNr) in patients with Parkinson's disease (PD) as they made maximal voluntary grips. Relative to age-matched controls they had more rapid force decrement when contraction was meant to be sustained and prolonged release reaction time and slower rate of force offset when they were supposed to release the grip. These impairments were independent from medication status. Increased STNr power over 5–12 Hz (in the theta/alpha band) independently predicted better performance—reduced force decrement, shortened release reaction time and faster rate of force offset. In contrast, lower mean levels and progressive reduction of STNr power over 55–375 Hz (high gamma/high frequency) over the period when contraction was meant to be sustained were both strongly associated with greater force decrement over time. Higher power over 13–23 Hz (low beta) was associated with more rapid force decrement during the period when grip should have been sustained, and with a paradoxical shortening of the release reaction time. These observations suggest that STNr activities at 5–12 Hz and 55–375 Hz are necessary for optimal grip performance and that deficiencies of such activities lead to motor impairments. In contrast, increased levels of 13–25 Hz activity both promote force decrement and shorten the release reaction time, consistent with a role in antagonising (and terminating) voluntary movement. Frequency specific oscillatory activities in the STNr impact on motor performance from the beginning to the end of a voluntary grip.

## Highlights

► PD patients show impairments in maximal grip performance. ► Performance impairments correlate with subthalamic activity. ► Correlations differ according to frequency of subthalamic activity.

## Introduction

Maximal voluntary contractions demonstrate many of the motor impairments of PD. The development of force may be slow, peak force may be attenuated, force may wane more rapidly than normally and contraction offset may be delayed (Corcos et al., 1996; Gordon, 1998; Jordan et al., 1992; Kunesch et al., 1995; Ziv et al., 1998). Local field potential (LFP) recordings made from the basal ganglia of patients undergoing deep brain stimulation have suggested that the basal ganglia are involved in determining the rate of force development and the peak force developed early in the contraction (Anzak et al., 2012). Specifically, the level of theta/alpha and gamma band activity around the time of movement onset correlates with these aspects of initial performance. This is in line with growing evidence that the basal ganglia determine the force or vigour of a motor response (Turner and Desmurget, 2010).

However, the extent to which the basal ganglia might be involved in those motor impairments evident later in a maximal contraction, such as during a manual grip, is less clear. Here we relate LFP activities to difficulties in maintaining maximal force and in relaxing contraction on task termination in patients with PD. The rate of force decrement may be considered an aspect of bradykinesia, similar to the decrement in finger tapping that forms a hallmark of the condition (Ling et al., 2012). The difficulty in relaxing contraction has also been found to closely correlate with global measures of motor impairment (Corcos et al., 1996; Jordan et al., 1992; Kunesch et al., 1995). Our working hypothesis in the present study is that basal ganglia activities contribute to these aspects of performance and that this will be manifest in correlations between LFP activities and measures of force decrement and delayed offset. We also hypothesise that the same basal ganglia features might correlate with more than one aspect of motor performance and that such patterns of association might therefore allow inferences about the underlying function served by activities reflected in the basal ganglia LFP.

## Methods

We extended the analysis made in a previously published cohort of PD patients (Anzak et al., 2012) and age-matched healthy controls (Anzak et al., 2011b) to include behavioural measures of performance made later in a maximal voluntary grip and analysis of the corresponding LFP recorded in PD patients.

### Subjects

Ten patients with PD (mean disease duration 10 years, mean age 58 years, range 42–65 years; seven males) and ten age-matched healthy controls (mean age 60 years, range 41–73 years; seven males; with one new healthy control subject apart from those published in Anzak et al., 2011b) provided informed consent to take part in this study, which was approved by the local ethics committees. There was no significant difference between the ages of the two groups (*t*_18_ = 0.570, *p* = 0.576). The patients showed 59.9 ± 5.7% (*p* < 0.0001) improvement in the motor section of the Unified Parkinson's Disease Rating Scale (UPDRS) on treatment with levodopa (L-DOPA), indicating good responsiveness to L-DOPA. Patients underwent bilateral implantation of DBS electrodes into the STN, as a prelude to therapeutic high frequency stimulation for advanced idiopathic PD with motor fluctuations and/or dyskinesia. Other details about these patients have been previously reported (Anzak et al., 2012).

### Experimental paradigm

Subjects were seated with their shoulders adducted (so that elbows rested against the trunk), their elbows flexed at about 90° and their forearms in neutral, as recommended by the American Association of Hand Therapists (Fess, 1992). A series of imperative visual (V) cues (illumination of a red light-emitting-diode—LED) were presented to the subjects, and the subjects were instructed to squeeze a force dynamometer “as fast and hard as you possibly can when the light comes on, maintain this for the duration of the light and release the grip when the light turns off.” For each trial, the red LED was illuminated for 5 s and each trial was separated by 6–8 s rest. In half of these trials, randomly selected, a loud auditory stimulus (0.3 s duration, 1 kHz, 96 dB) was delivered binaurally through headphones, with onset simultaneous with that of the V cue (AV; auditory-visual cue). However, subjects were asked to just focus on responding to the V cues. The rationale for the number of trials executed and the inter-trial interval, as well as stimulus intensity has previously been described (Anzak et al., 2011a,b).

In patients, recordings were made 3–6 days after surgery. In order to complete the recordings in one morning, and limit intrusion on our easily fatigable post-operative patients, recordings were always made first after overnight withdrawal of anti-parkinsonian medication (OFF L-DOPA), and then again approximately 1 h after taking their usual morning dose (average L-DOPA dose administered, 155 ± (SEM) 25 mg, two patients also received subcutaneous apomorphine). This sequence of recordings may have introduced a confound, since on medication performance may have been affected by fatigue. Healthy controls were also asked to undertake two experimental runs, with a 45- to 60-min break in between, in order to match any practice, habituation or fatigue effects in the patients.

### Recordings

Grip force was measured one hand at a time in each subject using an isometric dynamometer (G200, Biometrics Ltd., UK). Bipolar LFPs were recorded from adjacent contacts of each DBS electrode (0–1, 1–2, 2–3) and pass band filtered between 0.5 and 500 Hz using either a D360 amplifier (Digitimer Ltd., Welwyn Garden City, Hertfordshire, UK) in combination with a 1401 A-D converter (Cambridge Electronic Design, Cambridge, UK) and sampled onto a computer using Spike2 software (Cambridge Electronic Design), or TMSi porti (TMS international, Netherlands) and its respective software. All recordings were originally sampled at 2048 Hz. Analogue correlates of the visual and auditory stimuli and dynamometer output were recorded and digitised in a similar way.

### Analysis

Analyses of both behavioural and LFP data were performed in Matlab (version 2010b). The grip force trajectory of each individual trial of each subject was first normalised against the maximal force each subject achieved in the V condition. The waning of force over the period during which maximal contraction was meant to be sustained was measured as the gradient (*k*) of the regression line fitting force over the period from peak force to the time of offset of the LED cue. Two parameters were derived to describe the force release on termination of grip: the release reaction time (R-RT) and the rate of force release (R-Rate). Release reaction time (R-RT) was operationally defined as the time interval between offset of the LED cue and onset of release of the contraction, with the latter defined as the time when grip force reduced to 90% of the average force over the one second before the offset of the cue. Releasing rate (R-Rate) was defined as the inverse of the time between the onset of the release of force and the point at which force reduced to 10% of the average force over the one second before the offset of the LED cue (see Supplementary Figure for schematic showing the definition of these measurements).

Notch filters (5th order zero-phase Butterworth filters) were used to remove the line noise artefacts at 50 Hz and 100 Hz. The average LFP across trials was subtracted from the original local field potential in each trial so that further analysis would estimate induced power. A time–frequency decomposition based on the continuous wavelet transform was then applied to each (average-subtracted) trial to analyse changes in induced LFP activity in the time–frequency domain. Event related LFP power was subsequently normalised by calculating the *z*-score of the power at each time point relative to the power between two seconds and one second before the cue, so that a value higher than zero indicated power higher than before cue and *vice versa*. The normalised induced power was aligned to cue presentation, and averaged across trials of a given type and subsequently across the three bipolar contacts for each STNr lead contralateral to the gripping hand. We averaged across all the contact pairs in a given electrode so as to avoid selection bias, although not all contacts will have been in the STN *per se*.

### Statistics

Grand averages of behavioural and LFP data for different experimental conditions were calculated after deriving each of these variables from the individual grips made by a subject, and calculating averages for that subject, and then averaging across study participants. For LFP measurements, significant differences from baseline for each condition were first evaluated using one-sample *t*-tests. Differences between conditions were assessed with analyses of variance (ANOVA). Means ± standard error of means (SEM) are presented throughout the text, unless otherwise specified.

Generalised linear models were used to identify which, if any, frequency specific LFP activities were significant independent predictors for motor parameters during maximal grip and its release. Modelling included experimental condition (Drug status and Stimulus type) as factor predictor, average normalised induced power in different frequency bands as covariance predictor, and any 2-way interaction between the experimental conditions and the LFP activities. Models were gradually simplified by removing those items that were not significant predictors and did not improve fitting of the model. The final models were evaluated using the Omnibus Test to see whether they accounted for significantly more variance in the dependent variables than models with only intercepts, and the *B* coefficient of each significant predictor reported. A *B* coefficient of 1.0 would indicate that for every unit increase in the predictor, the predicted value of the dependent variable also increased by one unit. Where there are two or more correlated predictors in the model, the *B* coefficient is known as a partial regression coefficient and it represents the predicted change in the dependent variable when that predictor is increased by one unit while holding all other predictors constant.

Statistical analyses were performed in SPSS Statistics 19 (SPSS Inc., Chicago, IL, USA). Kolmogorov–Smirnov tests were applied to confirm that behavioural measures and LFP data were normally distributed, prior to further parametric testing. Where Mauchly's test of sphericity was significant (*p* < 0.05) in repeated measures ANOVAs, Greenhouse–Geisser corrections were applied. Multiple comparisons were corrected for using the false discovery rate procedure (Curran-Everett, 2000) and only those *p* values remaining significant following this procedure are given.

## Results

### PD patients have more rapid force decrement over time

**Fig. 1:**
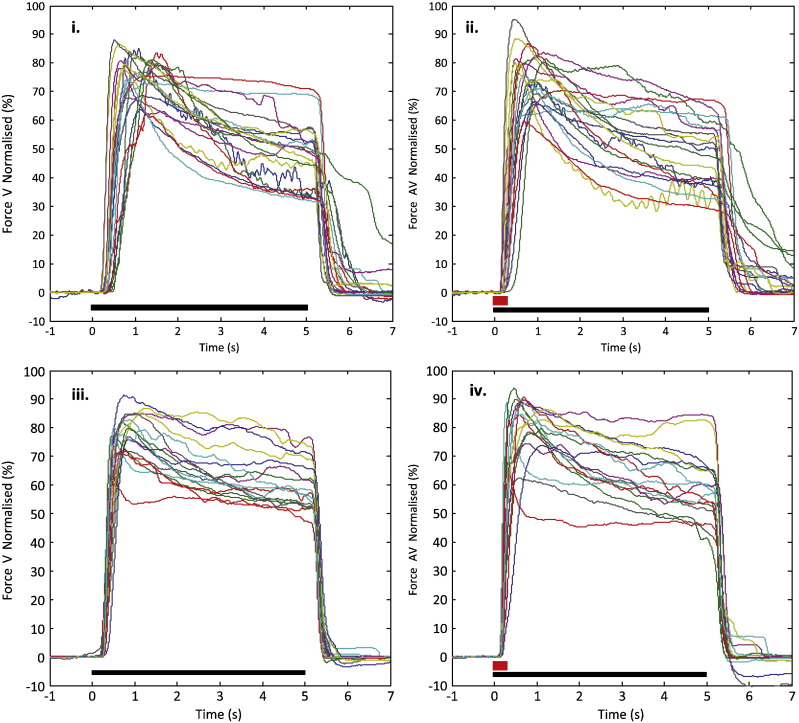
Average normalised grip forces after alignment to the cue onset in patients with Parkinson's disease ON L-DOPA when presented with visual cue only (i), when presented with additional auditory cue (ii) and the same for age-matched controls (iii and iv). Each line in a panel is the average force trajectory of one subject in that experimental condition and line colours are preserved across conditions for that subject. The black bar at the bottom of each plot indicates the duration of the LED light and the red bars at the bottom of panel iii and iv indicate a loud auditory cue with short duration.

We were interested in patients' behavioural performance under standard conditions (*i.e.* following V cues ON L-DOPA) and in departures from this performance under other conditions. Force decrement during the period over which contraction should have been sustained was estimated separately for each condition in each subject from the gradient, *k*, of the regression line fitting force over the period from peak force to the time of offset of the LED cue. Overall PD patients were less able to sustain peak force than age-matched healthy control subjects (Fig. 1).

A mixed design 2-way ANOVA with factors Group (PD patients and age-matched healthy control subjects) and Order (OFF L-DOPA in patients with PD/first experimental run in controls and ON L-DOPA in patients/second experimental run in controls) was applied to force decrement gradients in V trials. Force decrement was more rapid in PD (*k* = − 7.77 ± 0.63 percent/s for patients with PD, compared with *k* = − 4.71 ± 0.63 percent/s for healthy controls, *F*_1,38_ = 11.725, *p* = 0.001). The lack of effect of Order by itself (*F*_1,38_ = 2.451, *p* = 0.126), or Group × Order interaction (*F*_1,38_ = 1.161, *p* = 0.288) suggests that levodopa did not affect force decrement, or the difference between patients and control subjects. A further mixed design 2-way ANOVA with factors Group and Stimulus (V and AV) on data averaged across different drug states/experimental orders showed no effect of Stimulus (*F*_1,38_ = 0.126, *p* = 0.725) or interaction between Stimulus and Group (*F*_1,38_ = 1.319, *p* = 0.258).

### PD patients have impaired force release

Two parameters were derived to describe the release of force on termination of the grip: the release reaction time (R-RT) and the rate of force release (R-Rate). PD patients tended to be more delayed in the onset of grip release following the cue to terminate contraction, and the rate at which grip was released also tended to be slower than in age-matched healthy subjects (Fig. 1).

**Fig. 2:**
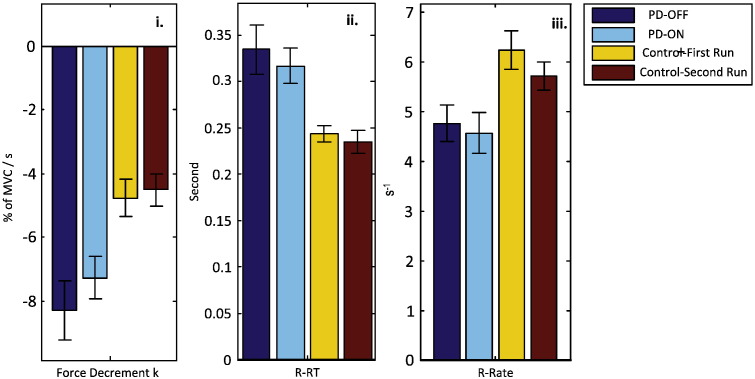
Behavioural difference between patients with Parkinson's disease and healthy controls during maximal grip and its release: patients had quicker force decrement during sustained contraction (i), slower releasing reaction time (ii), and slower rate of force release (iii) compared with age-matched healthy controls. Impairments were independent of drug status. Data collapsed across different cue types since there was no effect of cue type on performance during maximal grip or its release.

A mixed design ANOVA with factors Group and Order performed for V trials showed that patients with PD had significantly longer R-RT in response to releasing cues (326 ± 14 ms) compared with age-matched healthy controls (241 ± 14 ms) (*F*_1,38_ = 18.659 *p* < 0.001). This was independent from drug state, as indicated by a lack of main effect of Order (*F*_1,38_ = 0.845, *p* = 0.364), or of an interaction between Order and Group (*F*_1,38_ = 0.040, *p* = 0.843). The mean increase in R-RT for patients with PD was 35.3 ± 5.8% in V trials compared with healthy controls (Fig. 2).

R-Rate was 30.6 ± 6.9% slower in V trials in patients compared to controls (4.537 ± 0.317 s^− 1^ compared with 5.925 ± 0.313 s^− 1^, *F*_1,38_ = 9.816, *p* = 0.003) (Fig. 2). The R-Rate in PD patients was not affected by drug state (no effect of Order: *F*_1,38_ = 0.068, *p* = 0.796, and no Order × Group interaction: *F*_1,38_ = 1.135, *p* = 0.293).

A further mixed design ANOVA with factors Group and Stimulus (V and AV) on data averaged across different drug states/experimental runs showed no effect of Stimulus (*F*_1,38_ = 0.030, *p* = 0.864 for R-RT, and *F*_1,38_ = 0.072, *p* = 0.789 for R-Rate). There was also no interaction between Stimulus and Group (*F*_1,38_ = 2.287, *p* = 0.139 for R-RT and *F*_1,38_ = 1.641, *p* = 0.208 for R-Rate).

### Frequency specific activities in STNr during sustained contraction and active release

**Fig. 3:**
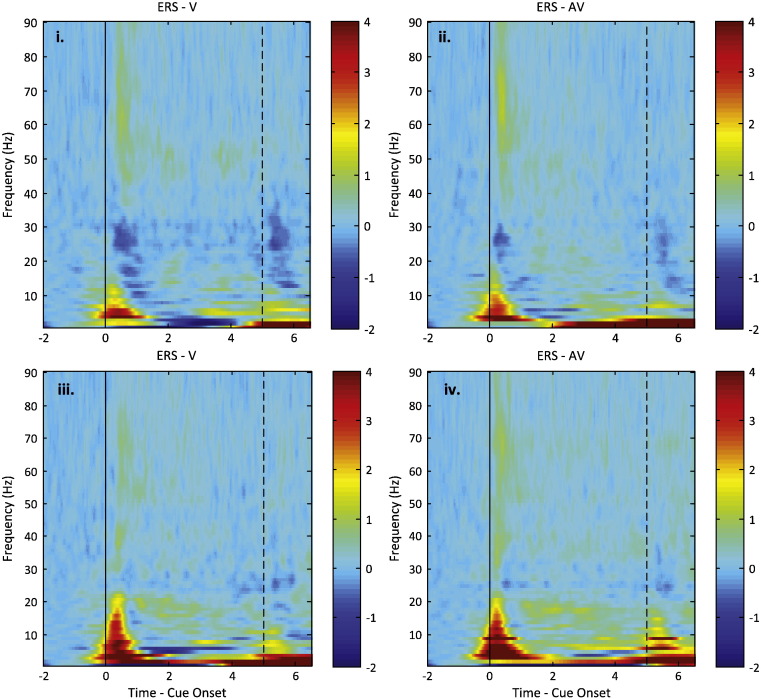
Average time–frequency plot of normalised induced spectral power (expressed as *z*-score relative to a pre-contraction baseline) in 20 subthalamic nuclei contralateral to sustained maximal handgrips under four different experimental conditions: i.) ON L-DOPA, visual (V) cue; ii.) ON L-DOPA, auditory-visual (AV) cue; iii.) OFF L-DOPA, visual cue; iv.) OFF L-DOPA, auditory-visual cue. Time zero represents onset of the LED light and the black dashed line indicates the turning off of the LED light.

Time-evolving power spectra of STNr LFPs relative to a pre-cue baseline were derived by averaging across all trials in an experimental condition, for each PD patient. These were then averaged across subjects (Fig. 3). We previously reported LFP changes from the onset of cue up until the generation of peak force (Anzak et al., 2012) and identified changes in five different frequency bands. These were the theta/alpha (5–12 Hz), low beta (13–23 Hz), high beta (25–30 Hz), low gamma (31–45 Hz) and high gamma/high frequency (55–375 Hz) ranges. Here we report average normalised power in these five different frequency bands during the sustained contraction phase (from the time to peak force to the time of LED offset) and during the grip release phase (from the time of offset of the LED cue to the point at which 10% of peak force was reached).

### LFP power during sustained contraction

We were interested in LFP responses under circumstances that were as physiological as possible and in departures from these responses under different conditions. So we first considered LFPs in V trials performed ON L-DOPA in patients with PD. One sample *t*-tests applied to the power in each of the five frequency bands relative to pre-contraction baseline only identified significant increases during the sustained contraction phase in the high gamma/high frequency band (*t*_19_ = 3.482, *p* = 0.002) following correction for multiple comparisons. A subsequent repeated measures ANOVA of relative high gamma/high frequency band power with factors Drug (ON/OFF L-DOPA) and Stimulus (V/AV) revealed no significant effects (Stimulus; *F*_1,19_ = 1.988, *p* = 0.175, Drug; *F*_1,19_ = 2.549, *p* = 0.127, Interaction between Stimulus and Drug: *F*_1,19_ = 0.259, *p* = 0.617).

### LFP power during active releasing

One Sample *t*-tests followed by false discovery rate procedure for the correction of multiple comparisons confirmed that there were significant changes in LFP power relative to pre-contraction baseline in all five frequency bands when patients were ON L-DOPA and presented with visual cues only. Power increased in the theta/alpha (*t*_19_ = 4.043, *p* = 0.001) and high gamma/high frequency (*t*_19_ = 2.474, *p* = 0.023) band and decreased in the low beta (*t*_19_ = − 2.871, *p* = 0.010), high beta (*t*_19_ = − 5.551, *p* < 0.001) and low gamma frequency (*t*_19_ = − 2.262, *p* = 0.036) bands.

**Fig. 4:**
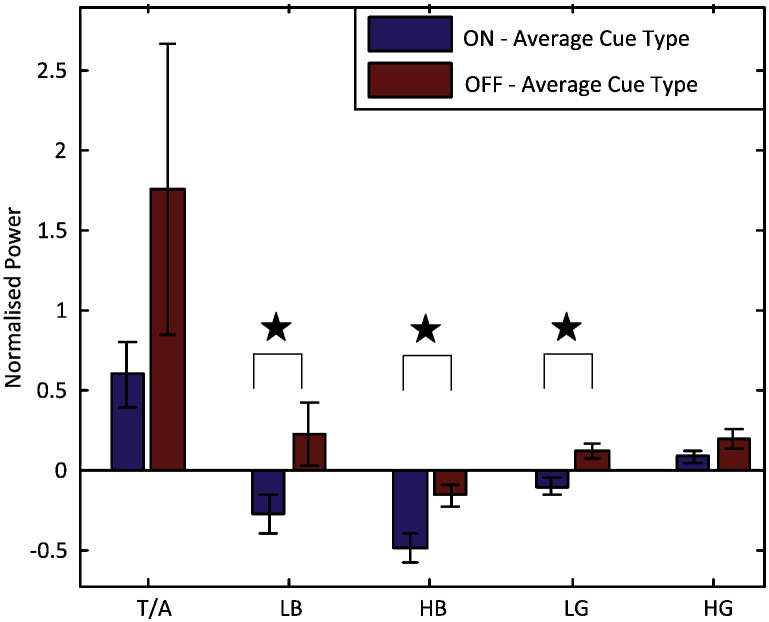
Mean ± SEM of normalised power (expressed as *z*-score relative to pre-contraction cue) of the five frequency bands during the release of maximal grip: power in theta/alpha band (T/A) and high gamma band (HG) increased and power in low beta (LB), high beta (HB) and low gamma (LG) frequency bands decreased compared with pre-contraction baseline when patients were ON L-DOPA. Powers in LB, HB and LG bands were significantly increased when the patients were OFF L-DOPA compared with ON L-DOPA. Data collapsed across different cue types since there was no effect of cue type or any interaction with cue across all the different frequency bands.

A repeated-measures ANOVA, with factors Drug (ON/OFF L-DOPA), Stimulus (V/AV) and Frequency (5 bands), was then applied to the average power relative to pre-contraction baseline. This identified an effect of Drug (*F*_1,19_ = 4.403, *p* = 0.049) and Frequency (*F*_1,19_ = 7.106, *p* < 0.001). There was no effect of Stimulus or any 2-way or 3-way interaction. Power was higher OFF than ON L-DOPA and this difference was significant in the low beta, high beta and low gamma bands (Fig. 4). Thus the task termination-related suppression of these three bands relative to baseline was diminished without L-DOPA.

### Frequency specific STNr LFP activity contributes to force decrement during sustained contraction

Generalised linear models were performed in SPSS with the aim of investigating the relationship between LFP power relative to pre-contraction baseline within the STNr and performance during maximal grip and its release. Drug status and stimulus type were included in the model as factor predictors.

**Fig. 5:**
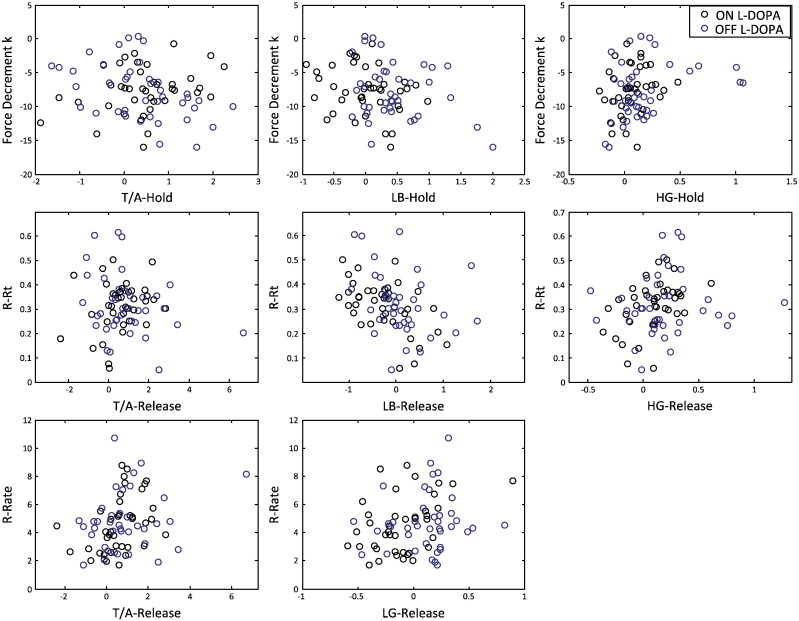
Behaviour during maximal grip (force decrement, *k*, units = % (of MVC) s^− 1^) and its release (R-Rt, units = s and R-Rate, units = s^− 1^) with respect to frequency specific LFP activities within the STNr identified as significant predictors. See legend to Fig. 4 for explanation of frequency band abbreviations.

The models identified normalised induced power in the low beta and high gamma/high frequency bands and an interaction between Drug and normalised theta/alpha power as significant and independent predictors of the force decrement during sustained contraction. Increased power in the high gamma/high frequency band (*B* = 4.44 ± 1.70, *χ*^2^ = 6.783, *p* = 0.009) and decreased power in the low beta band (*B* = − 1.903 ± 0.768, *χ*^2^ = 6.142, *p* = 0.013) were associated with less force decrement (Fig. 5). Thus, the increase in high gamma/high frequency activity relative to pre-cue baseline while force should have been sustained tended to protect against force decrement. The interaction between Drug and theta/alpha band power arose because force decrement decreased with increased power in the theta/alpha band (*B* = 1.775 ± 0.795, *χ*^2^ = 4.982, *p* = 0.026), but only when the patients were ON L-DOPA. The model including oscillatory activities explained significantly more variance in the data than the intercept by itself (Omnibus Test, likelihood ratio *χ*^2^ = 20.697, *df* = 4, *p* < 0.001).

Amongst the spectral features predicting force decrement, normalised power in the high gamma/high frequency band had over twice the effect of that in the low beta or theta/alpha bands and suggested that force would wane more much rapidly if a subject had a low overall level of high gamma/high frequency activity in their STNr during the period over which force should have been sustained. We therefore explored whether changes in normalised power in high gamma/high frequency band within this period also had a bearing on force, expecting progressive drops in such activity to relate to force decrement. We divided the contraction from the point at which peak force was first developed to the point at which the LED was extinguished into serial non-overlapping periods of 0.5 s duration. The force and normalised power in each of the five frequency bands were determined for each period from the mean data for that condition per subject. These were then correlated and the correlation coefficients Fisher transformed. ANOVA of transformed correlation coefficients with factors Frequency (5 bands), Drug (ON/OFF L-DOPA) and Stimulus (V/AV) confirmed a main effect of Frequency (*F*_4,76_ = 19.448, *p* < 0.001). This was due to correlation in the high gamma/high frequency band that, averaged across conditions and subjects, was 0.571 ± 0.060 and exceeded that in the remaining four frequency bands (*p* < 0.001 for all four paired *t*-tests). Thus, the more high gamma/high frequency activity dropped over time during the period when contraction should have been sustained the more force was likely to drop within a subject. Correlations between high gamma/high frequency activity and force decrement were significant in 39% of conditions across individual subjects. The correlation coefficient between high gamma/high frequency and force was not changed by Drug status (*F*_1,19_ = 0.234, *p* = 0.634) or Stimulus type (*F*_1,19_ = 0.003, *p* = 0.959), or any interaction between Drug status and Stimulus type (*F*_1,19_ = 3.285, *p* = 0.086).

### Frequency specific STNr LFP activity contributes to release reaction time and rate of force release

A generalised linear model with normalised power in the theta/alpha, low beta and high gamma/high frequency (but not low gamma bands) during grip release was highly significant in explaining the variance in R-RT (likelihood ratio *χ*^2^ = 20.229, *df* = 4, *p* < 0.001). The model indicated that R-RT reduced as normalised power in the theta/alpha and low beta bands increased (*B* = − 0.017 ± 0.0054, *χ*^2^ = 10.032, *p* = 0.002 for theta/alpha and *B* = − 0.101 ± 0.024, *χ*^2^ = 17.734, *p* < 0.001 for low beta; Fig. 5). In contrast, R-RT increased with an increase in high gamma/high frequency (*B* = 0.087 ± 0.0418, *χ*^2^ = 4.317, *p* = 0.038; Fig. 5). The effect on R-RT of proportionally similar changes amongst these three power bands was by far the weakest in the theta/alpha band. The addition of either Drug or Stimulus type or any 2-way interaction as predictors did not improve the model.

The rate of force release was explained by a generalised linear model, with normalised power in the low gamma band and an interaction between Drug and normalised alpha power (likelihood ratio *χ*^2^ = 10.863, *df* = 3, *p* = 0.012). The rate of force release increased with increased low gamma power (*B* = 1.803 ± 0.734, *χ*^2^ = 6.036, *p* = 0.014) and, when ON L-DOPA, increased theta/alpha power (*B* = 0.600 ± 0.263, *χ*^2^ = 5.233, *p* = 0.022).

## Discussion

We have previously shown that frequency-specific STNr LFP activities can explain much of the variance in different measures related to grip onset and here we show this for later aspects of grip performance (Table 1). Below, we will consider in more detail the predictive value of LFP activities in different frequency bands. However, first we should highlight a negative result from our study. We examined maximal grips prompted by both simple visual cues and such cues accompanied by a short high intensity sound. However, stimulus type neither affected behaviour nor LFP power later in the contraction, after peak force was reached. We previously reported a relatively minor effect of AV cuing limited to the high gamma/high frequency band immediately after the imperative cue instructing grip onset (Anzak et al., 2012). The present findings would suggest that this effect of AV cuing on the LFP is short-lived.

### Theta/alpha activity

Theta/alpha activity correlated with force measures during the period when contraction should have been sustained and during grip release, and with the latency to onset of grip release. The directionality of these correlations, like those established with performance earlier in the contraction (Anzak et al., 2012), was consistent with higher levels of theta and alpha power in the STNr facilitating normal behaviour. The breadth of these associations, which included both force and reaction time measures, raises the possibility that activity in the theta/alpha band might subserve a non-specific function related to movement, such as attention. This would be in line with the general association of oscillatory activity in this frequency band with mechanisms of attention (for review see Palva and Palva (2011)) and with views about the coherent activity at similar frequencies between the STNr and parieto-temporal cortex of patients with PD (Hirschmann et al., 2011; Litvak et al., 2011). Singh et al. (2011) have suggested that alpha synchronisation in the basal ganglia may be primarily physiological rather than disease specific, as they demonstrated increases in alpha activity during fast movements compared to rest in both the STNr of patients with PD and the globus pallidus interna of patients with dystonia.

### Beta activity

Elevated beta activity in the basal ganglia has been associated with Parkinsonism (Jenkinson and Brown, 2011), and here we found that increased power in the low beta band promoted force decrement over time. Nevertheless, the effect of beta activity on force decrement was rather less than that of high gamma/high frequency activity and there were no other associations between raised beta power and impaired function in the grip task (Table 1). This is surprising as robust correlations have been reported between treatment induced suppressions of beta activity and improvements in bradykinesia and rigidity (Kühn et al., 2006, 2008, 2009; Ray et al., 2008; Weinberger et al., 2006). The explanation to this paradox may lie in the multivariate approach taken here (and in Anzak et al., 2012), which highlights those LFP features that continue to predict behavioural performance while other features are held constant. Thus the relationship hitherto reported between beta activity and bradykinesia-rigidity might be secondary to effects at lower and higher frequencies. This possibility would be in line with the hypothesis that beta suppression serves a binary gating function, whereby suppression allows task-related processing to occur with associated changes in oscillatory activities at other frequencies (Anzak et al., 2012; Brücke et al., 2012; Kempf et al., 2007).

Indeed, there was even a paradoxical improvement in performance upon grip release in the setting of higher levels of low beta power, when R-RT was reduced. Increases in beta activity have been associated with motor suppression, as in NoGo and stop-signal tasks, and it is conceivable that the hypothesised anti-kinetic properties of beta activity might play a role in grip termination (Kühn et al., 2004; Ray et al., 2012). As with alpha activity, movement-related reactivity of basal ganglia LFP activity in the beta band may be a physiological feature, as it can be detected in healthy monkeys (Courtemanche et al., 2003) and in non-parkinsonian patients (Androulidakis et al., 2007; Sochurkova and Rektor, 2003).

### High gamma/high frequency activity

Lower mean levels and progressive reduction of high gamma/high frequency power during the period when force should have been sustained were both strongly associated with greater force decrement over time. The latter was more marked in our patients than in healthy age-matched controls and may be considered a feature of bradykinesia (Ziv et al., 1998). The relationship with force was in keeping with the positive correlation between high frequency activity in the basal ganglia and force at the outset of the maximal grip (Anzak et al., 2012) and with the prokinetic characterization of such activity (Brown, 2003; Brücke et al., 2012; Foffani et al., 2003; López-Azcárate et al., 2010; Özkurt et al., 2011). The weaker relationship between high gamma/high frequency activity and R-RT could then be an indirect attentional one: if the subject concentrates on maintaining a strong contraction with correspondingly elevated levels of high gamma/high frequency activity, then they may be distracted by this effort and release RT prolonged.

Whether STNr activity in the high gamma/high frequency band has a primarily physiological role in determining the force or vigour of contraction is unclear (Jenkinson et al., in press). High gamma (35–100 Hz) activity in the globus pallidus interna (GPi) LFP has recently been implicated in the scaling of movement in patients with segmental dystonia (Brücke et al., 2012). A similar role has been suggested for gamma (60–90 Hz) synchronisation in the motor cortex of healthy subjects (Muthukumaraswamy, 2010), and this cortical activity is likely related to that at subcortical levels (Litvak et al., 2012). LFP activities at frequencies over 100 Hz have so far only been reported in the basal ganglia of patients with PD (Foffani et al., 2003; López-Azcárate et al., 2010; Özkurt et al., 2011), and so it impossible to say whether these may be physiological or not.

### Caveats and concluding remarks

One of the unexpected findings in our investigation was the lack of an effect of levodopa on behavioural performance in the grip task. This was despite the fact that treatment did improve LFP power suppression relative to pre-cue baseline in the release phase of the grip. The lack of behavioural change may have been due to the fixed order in which drug states were tested. Specifically, any improvement due to L-DOPA may have been offset by fatigue as on-drug recordings were always performed second. That said, not all studies have found improvements in grip measures following treatment with L-DOPA (Gordon, 1998; Jordan et al., 1992).

Thus, our patients had impaired performance and did not improve with treatment. We must therefore be circumspect in how we interpret the LFP-behaviour correlations; whether they relate to essentially physiological functions or are primarily pathological remains to be established. In the above discussion we have furnished limited circumstantial evidence that changes in basal ganglia activity in each of the major frequency bands can be physiological but whether the degree of reactivity is similar to that occurring under physiological situations remains entirely presumptive.

Finally, we should consider whether the power changes picked up at the bipolar contacts of the DBS electrode could be the product of volume conduction from another source, possibly cortical, rather than locally generated. Against this, we previously reported a steep gradient in LFP power between those bipolar contact pairs recording the highest absolute power and the two remaining contact pairs, consistent with a local generator in this patient cohort (Anzak et al., 2012). Also of note, a number of studies have now demonstrated the locking of the discharge of neurons in the STN to LFP oscillations over a wide frequency band (Kühn et al., 2005; Levy et al., 2002; Pogosyan et al., 2006; Trottenberg et al., 2006; Weinberger et al., 2006).

In summary, we have shown that a range of performance measures that relate to bradykinesia in a maximal grip task was associated with reduced levels of theta/alpha and high gamma/high frequency activity and to a lesser extent, with elevation of beta activity in the STNr. The basal ganglia have no direct motor function and yet the present findings suggest that oscillatory activity within this system may have considerable and widespread effects on behavioural performance, even within a simple manual grip.

The following are the supplementary data related to this article.

Supplementary Figure Definition of performance measurements during maximal grip and its release: Force decrement *k* is defined as the slope of the regression line (red dashed line) fitting force over the period from peak force to the time of offset of the LED cue (black dashed line). Release reaction time (R-Rt) is the time interval between the offset of the LED cue and onset of release of the contraction, with the latter defined as the time when grip force reduced to 90% of the average force over the one second before the offset of the cue (red star). Releasing rate (R-Rate) was defined as the inverse of the time between the onset of the release of force and the point at which force reduced to 10% of the average force over the one second before the offset of the LED cue, which is shown as the red circle.

## Figures and Tables

**Table 1:** Relationship between STNr LFP activities in different frequency bands and motor performance in different phases of maximal voluntary contraction. Lower levels of theta/alpha power and high gamma/high frequency power were associated with poorer performance, with the exception that less high gamma/high frequency power was associated with shortened reaction time during release (R-RT). Higher power levels in the low beta band were associated with greater force decrement during the period when grip force should have been sustained, and a paradoxical improvement in R-RT upon release. *Results drawn from Anzak et al., 2012.

Bradykinesia measured in different phases	Grip onset *			Grip holding	Grip release	
	Longer onset RT	slower force development	Reduced peak force	Greater decrement	Longer R-RT	Slower R-Rate
Theta/alpha	↓	↓	↓	↓ON only	↓	↓ ON only
Low beta				↑	↓	
High beta						
Low gamma						↓
High gamma		↓	↓	↓	↑	
