# Supplementary material for: Frequency specific activity in subthalamic nucleus correlates with hand bradykinesia in Parkinson's disease
Source: Exp Neurol. 2013 Feb;240:122–9. doi: 10.1016/j.expneurol.2012.11.011 (PMC3605592; doi:10.1016/j.expneurol.2012.11.011)
Supplement: Supplementary Figure — Definition of performance measurements during maximal grip and its release: Force decrement k is defined as the slope of the regression line (red dashed line) fitting force over the period from peak force to the time of offset of the LED cue (black dashed line). Release reaction time (R-Rt) is the time interval between the offset of the LED cue and onset of release of the contraction, with the latter defined as the time when grip force reduced to 90% of the average force over the one second before the offset of the cue (red star). Releasing rate (R-Rate) was defined as the inverse of the time between the onset of the release of force and the point at which force reduced to 10% of the average force over the one second before the offset of the LED cue, which is shown as the red circle. [file mmc1.pdf]

Supplementary Figure

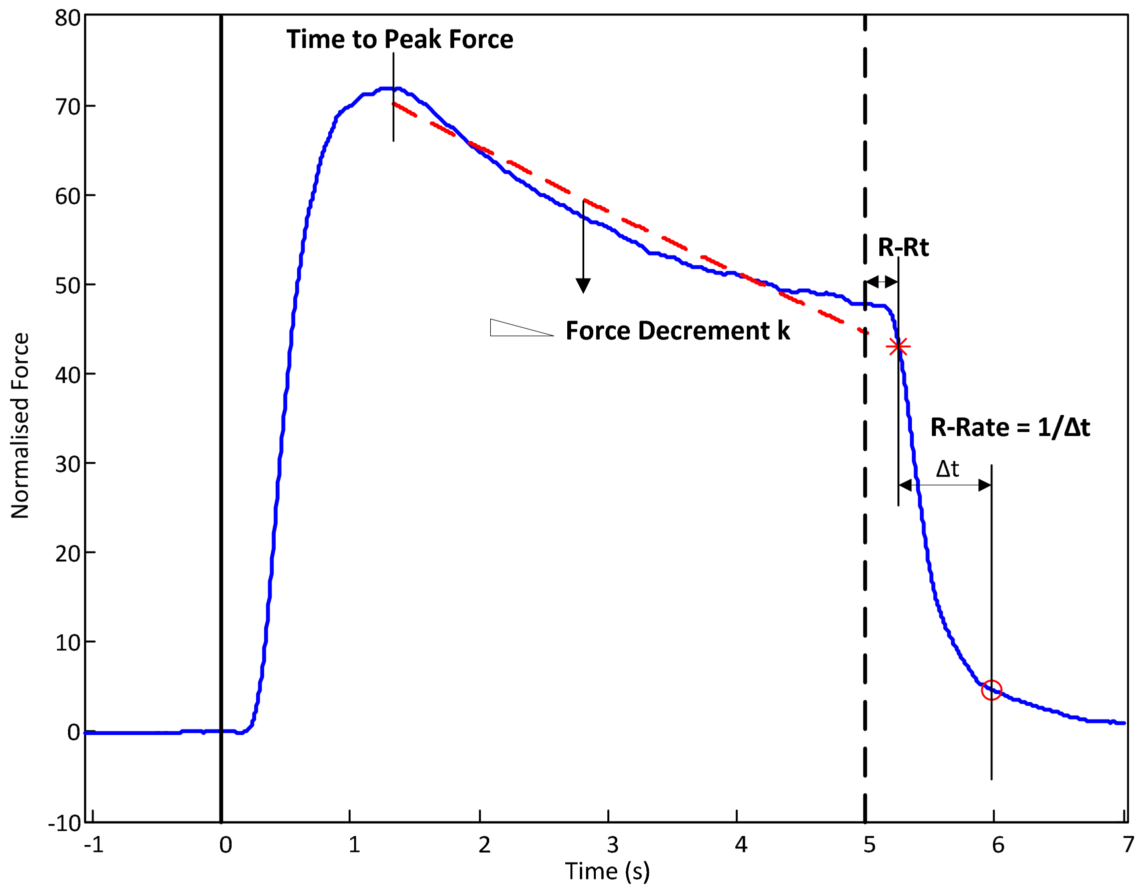

Supplementary Figure: Definition of performance measurements during maximal grip and its release: Force Decrement k is defined as the slope of the regression line (red dashed line) fitting force over the period from peak force to the time of offset of the LED cue (black dashed line); Release reaction time (R-Rt) is the time interval between the offset of the LED cue and onset of release of the contraction, with the latter defined as the time when grip force reduced to 90% of the average force over the one second before the offset of the cue (red star). Releasing rate (R-Rate) was defined as the inverse of the time between the onset of the release of force and the point at which force reduced to 10% of the average force over the one second before the offset of the LED cue, which is shown as the red circle.
